# Inhibition of PCAF Histone Acetyltransferase, Cytotoxicity and Cell Permeability of 2-Acylamino-1-(3- or 4-Carboxy-phenyl)benzamides

**DOI:** 10.3390/molecules171113116

**Published:** 2012-11-05

**Authors:** Woong Jae Park, Eunsook Ma

**Affiliations:** College of Pharmacy, Catholic University of Daegu, Hayang 712-702, Korea

**Keywords:** histone acetyltransferase (HAT), p300/CBP associated factor (PCAF), cytotoxicity, 2*-*acylaminobenzamide, Caco-2 cell permeability

## Abstract

Small molecule HAT inhibitors are useful tools to unravel the role of histone acetyltransferases (HATs) in the cell and they also have relevance in oncology. We synthesized a series of 2-acylamino-1-(3- or 4-carboxyphenyl)benzamides **8**–**19** bearing C6, C8, C10, C12, C14, and C16 acyl chains at the 2-amino position of 2-aminobenzoic acid. Enzyme inhibition of these compounds was investigated using *in vitro* PCAF HAT assays. The inhibitory activities of compounds **8**–**10**, **16**, and **19** were similar to that of anacardic acid, and **17** was found to be more active than anacardic acid at 100 μM. Compounds **11**–**15** showed the low inhibitory activity on PCAF HAT. The cytotoxicity of the synthesized compounds was evaluated by SRB (sulforhodamine B) assay against seven human cancer cell lines: HT-29 (colon), HCT-116 (colon), MDA-231 (breast), A549 (lung), Hep3B (hepatoma), HeLa (cervical) and Caki (kidney) and one normal cell line (HSF). Compound **17** was more active than anacardic acid against human colon cancer (HCT 116, IC_50_: 29.17 μM), human lung cancer (A549, IC_50_: 32.09 μM) cell lines. **18** was more active than anacardic acid against human colon cancer (HT-29, IC_50_: 35.49 μM and HCT 116, IC_50_: 27.56 μM), human lung cancer (A549, IC_50_: 30.69 μM), and human cervical cancer (HeLa, IC_50_: 34.41 μM) cell lines. The apparent permeability coefficient (P_app_, cm/s) values of two compounds (**16** and **17**) were evaluated as 68.21 and 71.48 × 10^−6^ cm/s by Caco-2 cell permeability assay.

## 1. Introduction

Epigenetics refers to the study of cellular phenotype changes without causing alteration of the genotype [1]. Epigenetic factors in human disease were first detected in 1983 by Feinberg and Vogelstein [2]. To date the majority of diseases in which epigenetic defects have been shown to be involved in disease pathogenesis are cancers [3]. The concept of the molecular aspects of epigenetics is rapidly evolving. Three molecular mechanisms, DNA methylation, histone modification and RNA-associated silencing, which are known to interact with each other, have been shown to be involved. 

Eukaryotic DNA is intimately associated with a family of small, basic histone proteins to form a highly ordered and condensed DNA-protein complex termed chromatin [4]. Recent studies implicated alteration in chromatin structure by histone hyperacetylation/deacetylation, are important in eukaryotic gene transcription, carcinogenesis, and cancer therapy. The dynamic equilibrium between acetylation and deacetylation is maintained by the activity of histone acetyltransferases (HATs) and histone deacetylases (HDACs) that regulate the expression of the genome. Specially, HATs function enzymatically by transferring an acetyl group from acetylcoenzyme A to the ε-amino group of certain lysine side chains within a histone’s basic *N*-terminal tail region [5]. Translation, amplication, overexpression or mutation of HAT gene occurs in a variety of cancers, especially those of epithelial origin [6]. 

HATs are divided into five families, including the GNAT family [GCN, PCAF (p300/cyclic AMP-responsive element binding protein associated factor))] [7], the MYST (named for members MOZ, Ybf/Sas3, Sas2 and Tip60) family and the p300/CBP HATs family [8], the general transcript factor, and the nuclear hormone-related HATs [9]. The PCAF HAT is the most thoroughly studied among different HAT families. It is a highly potent enzyme that acetylates histones and several other proteins.

Disfunction of HAT leads to several diseases including cancer, diabetes, viral infection and asthma [10], therefore, small molecule inhibitors are being considered as potential new generation therapeutics. Very few small molecules and cell permeable inhibitors for GCN5 and PCAF have been described until now. The first report of HAT inhibitors involved the design and synthesis of peptides conjugated with acetyl-CoA, including Lys-CoA that selectively inhibits p300 and H3-CoA-20 that is selective for PCAF [11]. Garcinol is a polyprenylated benzophenone analog that potently inhibit p300 or PCAF HAT inhibitors [12]. Curcumin was reported as p300/CREB-binding protein-specific cell permeable inhibitor of acetyltransferase [13]. Isothiazolone derivatives were also disclosed as inhibitors of PCAF and showed cell-permeability, but they exhibit significant off target activity *in vivo* due to their high chemical reactivity with thiol groups [14].

**Figure 1:**
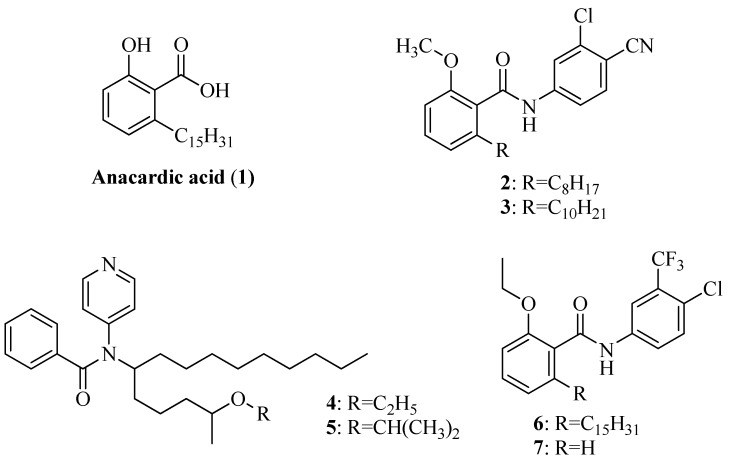
HAT inhibitors (**1**–**5**) and activator (**6** and **7**).

Anacardic acid (**1**, Figure 1), a bioactive phytochemical found in the nutshell of *Anacardium occidentale*, was found to be a compelling non-competitive inhibitor of p300, PCAF and MYST family HAT member Tip60 [15]. Even though anacardic acid does not affect DNA transcription directly, HAT-dependent transcription from chromatin template was strongly inhibited by anacardic acid which also induced cytotoxicity towards several human cancer cell lines *in vitro*. Futhermore, treatment of human cancer cells with anacardic acid increased their sensitivity to ionizing radiation, providing a novel therapeutic approach to radiosensitization of tumor cells, but anacardic acid was found to display poor cell permeability, thus limiting its practical applications [16].

With the purpose of improving the cell permeability, it was proposed to synthesize the potent benzamide derivatives from an anacardic acid derivatives and test them for anticancer activity. Cyanobenzamide derivatives **2** and **3** acted as cell-permeable inhibitors of p300, exhibiting a similar profile to anacardic acid [17]. Chandregowda *et al.* reported that the synthesis of benzamide derivatives of anacardic acid and 2-isopropoxy- and 2-ethoxy-6-pentadecyl-*N*-pyridin-4-yl benzamides (compounds **4** and **5**, Figure 1) were comparable with garcinol which is a cell permeable HAT inhibitor on HeLa cell line [18]. Interestingly, two benzamide derivatives *N*-(4-chloro-3-trifluoromethylphenyl)-2-ethoxy-6-pentadecylbenzamide (CTPB, **6**) and *N*-(4-chloro-3-trifluoromethyl-phenyl)-2-ethoxybenzamide (CTB, **7**) exhibited activation of the p300 HAT activity [19] (Figure 1). On the basis of the knowledge gained from its HAT inhibitory activity, the chemical formula of anacardic acid has been used for development of new synthetic HAT inhibitors and activators [20].

The anticancer effects of HDAC inhibitors are well known and a number of them are in clinical trials, while the chemotherapeutic potential of HAT targets has been less validated. New specific inhibitors would help to elucidate molecular mechanisms of the action of HATs in cells. Therefore, a facile synthetic method, new skeleton structures and good cell permeability are essential for developing a variety of HAT inhibitors. Among the wide variety of synthetic compounds recognized as potential anticancer drugs, molecules based on the 2-aminobenzoic acid (anthranilic acid) scaffold have attracted great interest in recent years. With the aim of establishing more facile synthetic methods and possibly improving the inhibitory activity and cell permeability, we replaced the hydrophobic long alkyl chain of anacardic acid with the long *N*-acyl chain at the 2-position of 2-aminobenzoic acid and introduced 3- or 4-carboxyphenylamino group at the 1-position of 2-acylaminobenzoic acid instead of the carboxyl group of anacardic acid. To investigate the relationships between HAT inhibitory effect and cytotoxicity, synthesized compounds were evaluated the PCAF HAT inhibitory effects and the cytotoxic effects against cancer cell lines by SRB method. Cell permeability of synthesized compounds is evaluated by using carcinoma colon 2 (Caco-2) cell lines.

## 2. Results and Discussion

### 2.1. Synthesis

**Scheme 1:**
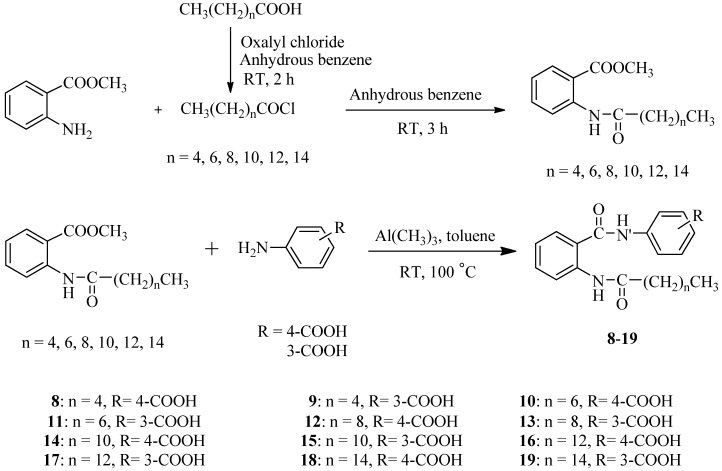
Synthetic method for compounds **8**–**19**.

Anacardic acid was used as the template for design and synthesis of several analogues, however these routes for synthesis of anacardic acid derivatives require harsh conditions that are not compatible with many functional groups [21,22,23]. To obtain the facile synthetic route for the diverse compounds displaying HAT inhibitory activity, we selected 2-aminobenzoic acid as starting material. A series of 2-acylamino-1-(3- or 4-carboxyphenyl)benzamides **8**–**19** was thus synthesized using procedures shown in Scheme 1.

2-Aminobenzoic acid was treated with six long chain alkanoic acid chlorides (C6, C8, C10, C12, C14, and C16), which were prepared from the corresponding acid with oxalyl chloride, to form six 2-acylaminobenzoic acids. The first attempts to synthesize 2-acylamino-1-carboxyphenylbenzamides by the reaction of 2-acylaminobenzoyl chlorides and 4- or 3-aminobenzoic acid were unsuccessful; it was thought this is due to the fact that the 2-acylaminobenzoic acid chlorides preferentially yielded benzoxazinone derivatives via intramolecular cyclization. The amide formation reaction of 2-acylaminobenzoic acid with 4- or 3-aminobenzoic acid in the presence of dicyclohexylcarbodiimide (DCC) or 1-ethyl-3-(3-dimethylaminopropyl)carbodiimide (EDCI) also failed to yield the desired benzamides. 

An alternative method for the MeAl_3_-mediated conversion of esters to amides has been reported [24]. Six acid chlorides were reacted with methyl anthranilate to give six methyl *N*-acylanthranilates in moderate to high yields. Compounds **8**–**19** were successfully synthesized from the reactions of six methyl 2-acylaminobenzoates with 3- or 4-aminobenzoic acid in the presence of MeAl_3_ [25]. 

**Figure 2:**
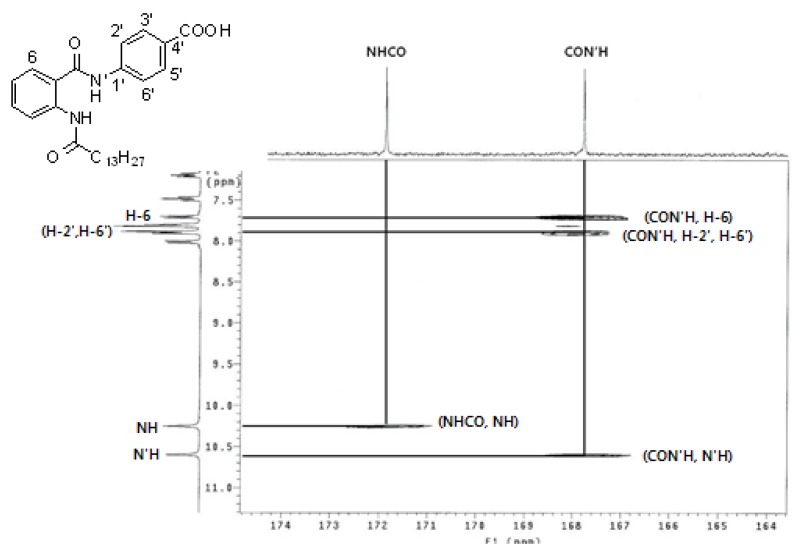
^1^H-^13^C HMBC spectrum of **16** (CDCl_3_).

All synthesized compounds were identified by ^1^H-NMR, ^13^C-NMR, ^1^H-^13^C HMBC, IR and mass spectroscopy. For example, two amide carbonyl group carbon peaks in **16** were determined by the ^1^H-^13^C HMBC spectrum as there were correlation between H-6 (δ 7.71 ppm) and H-2',6' (δ 7.90 ppm) with the carbon peak of CON'H (δ 167.8 ppm), and between N'H (δ 10.69 ppm) and the carbon peak of CON'H (δ 167.8 ppm) (Figure 2). Therefore, the carbonyl peak of CON'H is determined as 167.8 ppm and the carbonyl peak of NHCO is determined as 171.9 ppm.

### 2.2. PCAF HAT Inhibition Assay

**Figure 3:**
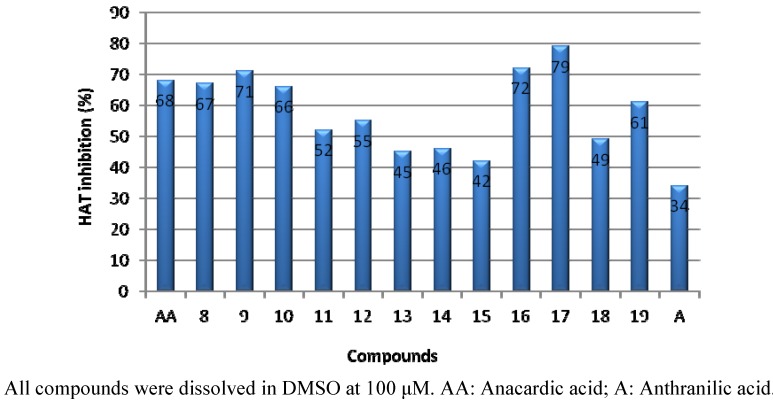
PCAF HAT inhibitory activity of **8**–**19** in 100 μM.

The inhibitory activity (%) of each compound was determined at 100 μM concentration (Figure 3). 2-Hexanoylamino-1-(4-carboxyphenyl)benzamide (**8**, 67%), 2-hexanoylamino-1-(3-carboxyphenyl) benzamide (**9**, 71%), 2-octanoylamino-1-(4-carboxyphenyl)benzamide (**10**, 66%), 2-hexanoylamino-1-(3-carboxyphenyl)benzamide (**11**, 71%), 2-tetradecanoylamino-1-(4-carboxyphenyl)benzamide (**16**, 72%), and 2-hexadecanoylamino-1-(3-carboxyphenyl)benzamide (**19**, 61%) showed similar inhibitory activity to AA (68%), but anthranilic acid (34%) showed low inhibitory activity to the enzyme.

Among the synthesized compounds, 2-tetradecanoylamino-1-(3-carboxyphenyl)benzamide (**17**, 79%) is more active than anacardic acid. Compounds **11**–**15** and **18** showed low inhibitory activity. These results indicate that the long 2-acylamino substituents of the benzamide analogs play an important role in the inhibitory activity and the 2-aminobenzoic acid scaffold can be used instead of salicylic acid in the anacardic acid. The PCAF HAT inhibitory activity was not dependent on the length of acyl chain at the 2-position and the introduction of 3-carboxy- or 4-carboxyphenylamino groups at the 1-position of 2-aminobenzoic acid did not contribute to increase the inhibitory activity, but the presence of 2-acylamino side chain is seemed to be decisive for inhibitory activity in comparison with anthanilic acid.

### 2.3. Cytotoxic Activity by SRB Assay

HAT inhibition data that have been accumulated over the last decade clearly link carcinogenesis and tumor progression to the deregulation of enzymes responsible for epigenetic modifications. Therefore, we investigated the cytotoxicity of cancer cell lines in order to explore the relationship between HAT inhibitory activity and cytotoxicity of 2-acylamino-1-(3- or 4-carboxyphenyl)benzamide analogs.

A series of 2-acylamino-1-(3- or 4-carboxyphenyl)benzamides **8**–**19**, with different length C6, C8, C10, C12, C14 and C16 acyl chains at the 2-position and introducing 3- or 4-carboxyphenylamino groups at the 1-position using 2-aminobenzoic acid as a synthon, were evaluated against seven cancer cell lines: HT-29 (human colon cancer), HCT-116 (human colon cancer), MDA-231 (human breast cancer), A549 (human lung cancer), Hep3B (human hepatoma cancer), HeLa (human cerviacel cancer) and Caki (human kidney cancer), and one normal cell line by SRB colorimetric assay, using an anacardic acid as the reference compound (Table 1). 

Compound **17** (n = 14, 3-carboxyphenyl) showed moderate cytotoxic activity against human colon cancer (HCT 116, IC_50_: 29.17 μM) and human lung cancer (A549, IC_50_: 32.09 μM) cell lines. **18** (n = 16, 4-carboxyphenyl) showed moderate level of cytotoxic activity against human colon cancer (HT-29, IC_50_: 35.49 μM and HCT 116, IC_50_: 27.56 μM), human lung cancer (A549, IC_50_: 30.69 μM), and human cervical cancer (HeLa, IC_50_: 34.41 μM) cell lines. Compound **16** (n = 14, 4-carboxyphenyl) displayed IC_50_ values of 43.90 and 46.18 μM against HCT 116 and A549 cell lines and **19** (n = 16, 3-carboxyphenyl) displayed IC_50_ values of 36.92, 40.27, 40.52, 43.82 and 44.76 μM against HeLa, HCT-116, A549, Hep3B and HT-29 cancer cell lines. Compounds **16**–**19** (C14 and C16) were more active than anacardic acid against all cancer cell lines except human breast cancer cell line (MDA-231) and **8**–**15** (C6, C8, C10, and C12) and anthranilic acid were inactive against most of cancer cell lines. Therefore, a long acyl chain is necessary for cytotoxic activity. 

**Table 1:** IC_50_ values (μM) of synthesized compounds **8**–**19** against seven cancer cell lines ^a^.

	HT-29	HCT-116	MDA-231	A549	Hep3B	HeLa	Caki	HSF
**8**	>100	>100	>100	>100	>100	NT	>100	>100
**9**	>100	>100	>100	>100	>100	NT	>100	>100
**10**	>100	>100	51.83 ± 0.68	>100	>100	>100	69.49 ± 7.16	>100
**11**	>100	>100	>100	>100	>100	>100	>100	>100
**12**	>100	>100	>100	>100	>100	>100	63.75 ± 2.39	>100
**13**	>100	>100	>100	>100	>100	>100	>100	>100
**14**	>100	53.30 ± 2.05	>100	>100	>100	>100	>100	>100
**15**	>100	>100	>100	>100	>100	>100	>100	>100
**16**	54.79 ± 1.05	43.90 ± 2.39	79.11 ± 3.27	46.18 ±0.83	73.30 ± 9.61	56.53 ± 0.87	63.08 ± 0.21	>100
**17**	54.90 ± 3.49	29.17 ± 3.86	62.43 ± 0.58	32.09 ±1.36	57.52 ± 0.26	54.97 ± 3.14	55.88 ± 5.09	>100
**18**	35.49 ± 2.45	27.56 ± 0.28	>100	30.69 ± 2.41	39.10 ± 6.52	34.41 ± 2.19	50.29 ± 1.49	>100
**19**	44.76 ± 2.98	40.27 ± 4.30	>100	40.52 ± 3.59	43.82 ± 5.91	36.92 ± 3.76	82.62 ± 5.89	>100
**A**	>100	>100	>100	>100	>100	>100	>100	>100
**AA**	60.42 ± 6.36	96.31 ± 4.33	44.32 ± 4.20	70.76 ± 0.41	>100	NT	>100	>100

^a^ Determined by SRB assay. A: Anthranilic acid, AA: Anacardic acid. NT: No test; Values are average of at least three experiments ± standard error, conducted in triplicate sample for each concentration.

### 2.4. Caco-2 Cell Permeabilty

Caco-2 cell cultures are widely used as an *in vitro* model in drug absorption studies. The model is useful in determining roles played by various physical and biochemical barriers to drug absorption [26,27]. Caco-2 cells have many properties similar to those of the enterocytes of the small intestine. They contain active transport and efflux proteins. According to the FDA, Caco-2 cell cultures can be used as an *in vitro* model in bioavailability/bioequivalence testing of highly soluble drugs that permeate cell layers well [28], together with *in vitro* dissolution tests. 

Cumulative amounts of compounds transported across Caco-2 cell monolayers were calculated from concentrations measured in the receiver (basolateral) compartments. Apparent permeability coefficients, P_app_ (cm/s), were calculated as described previously [28]:

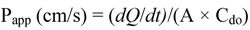

where *dQ/dt* is the rate of appearance of drug on the receiver side, A (cm^2^) is the surface area of the filter menbrane, and C_do_ is the initial drug concentration on the donor (apical) compartment. 

2-Tetradecanoylamino-1-(4-carboxyphenyl)benzamide (**16**) and 2-tetradecanoyl-1-(3-carboxyphenyl)-benzamide (**17**) as representative compounds were tested for permeability property and the P_app_ values were 68.21 and 71.48 × 10^−6^ cm/s (Table 2). Completely absorbed drugs were reported to have high permeability coefficients (Papp: >1 × 10^−6^ cm/s) [29], therefore this result suggest that the synthesized compounds, including **16** and **17**, will be completely absorbed in humans.

**Table 2:** P_app_ values of the two compounds.

	Initial Concentration (μM)	Measured P_app_ (×10^−6^ cm/s)
16	50	68.21 ± 3.56
17	50	71.48 ± 4.67

Results are mean values ± SD.

## 3. Experimental

### 3.1. General

All non-aqueous reactions were performed under an atmosphere of dry nitrogen. The commercial reagents were purchased from Aldrich, Fluka, or Sigma. Solvents were purified and dried prior to use. Melting points were measured on Thomas-Hoover melting point apparatus and not corrected. ^1^H-, ^13^C-NMR spectra were recorded on a Varian 400 MHz spectrometer in CDCl_3 _and DMSO-*d_6_*. Chemical shifts (δ) are in parts per million (ppm) relative to tetramethylsilane, and coupling constants (*J*) are in Hertz. IR spectra were determined on FT-IR JASCO 4100 spectrometer. GC/MS spectra were obtained on a Shimadzu QP 5050 and JEOL GC Mate 2 mass spectrometer. Analytical TLC was performed on pre-coated silica gel 60 F_254_ plates (Merck). Solvent systems for TLC were ethyl acetate/*n*-hexane mixtures and 10% MeOH in dichloromethane. Column chromatography was carried out on Merck silica gel 9385 (230–400 mesh). 

### 3.2. General Synthetic Methods

To a stirred solution of each of six alkanoic acid (C6, C8, C10, C12, C14 and C16_,_ 1.0 mmol) in anhydrous benzene (30 mL) was added oxalyl chloride (1.2 mmol) and the mixture was stirred at room temperature for 2 h. The excess benzene was evaporated to yield the corresponding alkanoic acid chloride, which was dissolved in anhydrous benzene (30 mL) and reacted with methyl 2-amino-benzoate at room temperature for 3 h. Water (30 mL) was added to the reaction mixture which was then was extracted with ethyl acetate (30 mL × 2). The combined organic extracts were dried with anhydrous MgSO_4_, filtered, and evaporated to yield methyl the 2-acylaminobenzoate analogs. These compounds (1.0 mmol) were dissolved in toluene and added with 4- or 3-aminobenzoic acid (1.2 mmol) and MeAl_3_ (2.0 M in toluene, 3.0 mmol) and stirred at room temperature for 1 h and then reacted at 100 °C for 1 h. The reaction mixture was cooled, treated with saturated NaHCO_3_ solution and extracted with ethyl acetate. The combined ethyl acetate solutions were dried with anhydrous MgSO_4_, filtered, and the filtrate evaporated to yield the crude compounds which were recrystallized or column chromatographed with ethyl acetate and *n*-hexane mixtures or methanol/dichloromethane mixtures to afford the pure compounds as a white or pale yellow powders.

*2-Hexanoylamino-1-(4-carboxyphenyl)benzamide* (**8**). The crude compound was recrystallized with ethyl acetate/*n*-hexane=1:1 mixture to give the pure compound as a white powder. Yield: 52%; mp: 216–218 °C; ^1^H-NMR (CDCl_3_) δ: 0.78 (3H, t, *J* = 6.6 Hz, CH_3_), 1.21–1.23 (4H, m, CH_2_ × 2), 1.52 (2H, quint, *J* = 14.4, 7.2 Hz, CH_2_), 2.26 (2H, t, *J* = 7.6 Hz, CH_2_), 7.20 (1H, dd, *J* = 7.6, 7.6 Hz, H-5), 7.49 (1H, dd, *J* = 7.8, 7.8 Hz, H-4), 7.70 (1H, d, *J* = 7.6 Hz, H-6), 7.81 (2H, d, *J* = 8.8 Hz, H-3', H-5'), 7.90 (2H, d, *J* = 8.4 Hz, H-2', H-6'), 8.00 (1H, d, *J* = 8.0 Hz, H-3), 10.22 (1H, s, NH), 10.60 (1H, s, N'H); ^13^C-NMR (CDCl_3_) δ: 14.5 (CH_3_), 22.5 (CH_2_), 25.4 (CH_2_), 31.4 (CH_2_), 37.4 (CH_2_), 120.2 (C-3', C-5'), 122.9 (C-3), 124.0 (C-5), 126.1 (C-1), 126.9 (C-1'), 129.4 (C-6), 130.8 (C-2', C-6'), 132.3 (C-4), 138.1 (C-2), 143.6 (C-4'), 167.8 (CON'H, COOH), 171.9 (NHCO); GC-MS (EI) *m/z*: 217 [M−1−C_7_H_6_NO_2_]^+^.

*2-Hexanoylamino-1-(3-carboxyphenyl)benzamide* (**9**). The crude compound was recrystallized with ethyl acetate/*n*-hexane = 1:1 mixture to give the pure compound as a pale yellow powder. Yield: 43%; mp: 219–221 °C; ^1^H-NMR (CDCl_3_) δ: 0.77 (3H, t, *J* = 5.4 Hz, CH_3_), 1.21–1.22 (4H, m, CH_2_ × 2), 1.53 (2H, quint, *J* = 14.4, 7.2 Hz, CH_2_), 2.27 (2H, t, *J* = 7.4 Hz, CH_2_), 7.19 (1H, dd, *J* = 7.8, 7.8 Hz, H-5), 7.41 (1H, dd, *J* = 8.0, 8.0 Hz, H-5'), 7.49 (1H, dd, *J* = 7.6, 7.0 Hz, H-4), 7.67 (1H, d, *J* = 7.2 Hz, H-6'), 7.74 (1H, d, *J* = 7.6 Hz, H-6), 7.87 (1H, d, *J* = 6.4 Hz, H-4'), 8.08 (1H, d, *J* = 7.6 Hz, H-3), 8.34 (1H, s, H-2'), 10.36 (1H, s, NH), 10.50 (1H, s, N'H); ^13^C-NMR (CDCl_3_) δ: 14.5 (CH_3_), 22.5 (CH_2_), 25.4 (CH_2_), 31.4 (CH_2_), 37.5 (CH_2_), 122.1 (C-2'), 122.6 (C-3), 123.8 (C-5), 125.0 (C-4'), 125.1 (C-6'), 125.3 (C-1), 125.5 (C-1'), 129.3 (C-6), 129.5 (C-5'), 132.3 (C-4), 138.4 (C-2), 139.7 (C-3'), 167.7 (CON'H, COOH), 171.9 (NHCO); GC-MS (EI) *m/z*: 217 [M−1−C_7_H_6_NO_2_]^+^.

*2-Octanoylamino-1-(4-carboxyphenyl)benzamide* (**10**). The crude compound was recrystallized with ethyl acetate/*n*-hexane = 1:2 mixture to give the pure compound as a white powder. Yield: 44%; mp: 192–194 °C; ^1^H-NMR (CDCl_3_) δ: 0.78 (3H, t, *J* = 7.0 Hz, CH_3_), 1.12–1.21 (8H, m, CH_2_ × 4), 1.51 (2H, quint, *J* = 14.4, 7.2 Hz, CH_2_), 2.26 (2H, t, *J* = 7.4 Hz, CH_2_), 7.20 (1H, dd, *J* = 7.6, 7.6 Hz, H-5), 7.49 (1H, dd, *J* = 7.6, 7.6 Hz, H-4), 7.70 (1H, d, *J* = 8.0 Hz, H-6), 7.81 (2H, d, *J* = 8.8 Hz, H-3', H-5'), 7.89 (2H, d, *J* = 8.4 Hz, H-2', H-6'), 7.98 (1H, d, *J* = 8.0 Hz, H-3), 10.21 (1H, s, NH), 10.59 (1H, s, N'H); ^13^C-NMR (CDCl_3_) δ: 14.6 (CH_3_), 22.7 (CH_2_), 25.7 (CH_2_), 29.1 (CH_2_ × 2), 31.8 (CH_2_), 37.4 (CH_2_), 120.2 (C-3', C-5'), 123.0 (C-3), 124.1 (C-5), 126.2 (C-1), 127.0 (C-1'), 129.4 (C-6), 130.8 (C-2', C-6'), 132.3 (C-4), 138.0 (C-2), 143.5 (C-4'), 167.8 (CON'H, COOH), 171.9 (NHCO); GC-MS (EI) *m/z*: 245 [M−1−C_7_H_6_NO_2_]^+^.

*2-Octanoylamino-1-(3-carboxyphenyl)benzamide* (**11**). The crude compound was column chromatographed with ethyl acetate/*n*-hexane = 1:1 mixtures and 10% MeOH in dichloromethane to give the pure compound as a white powder. Yield: 46%; mp: 189–191 °C; ^1^H-NMR (CDCl_3_) δ: 0.78 (3H, t, *J* = 6.8 Hz, CH_3_), 1.14–1.21 (8H, m, CH_2_ × 4), 1.52 (2H, quint, *J* = 14.4, 7.2 Hz, CH_2_), 2.27 (2H, t, *J* = 7.4 Hz, CH_2_), 7.19 (1H, dd, *J* = 7.6, 7.6 Hz, H-5), 7.42 (1H, dd, *J* = 8.0, 8.0 Hz, H-5'), 7.49 (1H, dd, *J* = 7.2, 7.2 Hz, H-4), 7.66 (1H, d, *J* = 7.6 Hz, H-6'), 7.74 (1H, d, *J* = 8.0 Hz, H-6), 7.90 (1H, d, *J* = 8.0 Hz, H-4'), 8.07 (1H, d, *J* = 8.4 Hz, H-3), 8.34 (1H, s, H-2'), 10.33 (1H, s, NH), 10.50 (1H, s, N'H); ^13^C-NMR (CDCl_3_) δ: 14.6 (CH_3_), 22.7 (CH_2_), 25.7 (CH_2_), 29.7 (CH_2_ × 2), 31.8 (CH_2_), 37.5 (CH_2_), 122.0 (C-2'), 122.7 (C-3), 123.5 (C-5), 124.4 (C-4'), 125.0 (C-6'), 125.4 (C-1, C-1'), 129.3 (C-6), 129.5 (C-5'), 132.3 (C-4), 138.3 (C-2), 139.7 (C-3'), 167.7 (CON'H, COOH), 171.9 (NHCO); GC-MS (EI) *m/z*: 245 [M−1−C_7_H_6_NO_2_]^+^.

*2-Decanoylamino-1-(4-carboxyphenyl)benzamide* (**12**). The crude compound was recrystallized with ethyl acetate/*n*-hexane = 1:1 mixture to give the pure compound as a white powder. Yield: 51%; mp: 196–198 °C; ^1^H-NMR (CDCl_3_) δ: 0.79 (3H, t, *J* = 6.6 Hz, CH_3_), 1.16–1.19 (12H, m, CH_2_ × 6), 1.51 (2H, quint, *J* = 13.6, 6.8 Hz, CH_2_), 2.26 (2H, t, *J* = 7.4 Hz, CH_2_), 7.19 (1H, dd, *J* = 7.6, 7.6 Hz, H-5), 7.48 (1H, dd, *J* = 8.0, 7.8 Hz, H-4), 7.72 (1H, d, *J* = 7.6 Hz, H-6), 7.78 (2H, d, *J* = 8.4 Hz, H-3', H-5'), 7.93 (2H, d, *J* = 7.6 Hz, H-2', H-6'), 8.05 (1H, d, *J* = 8.4 Hz, H-3), 10.33 (1H, s, NH), 10.59 (1H, s, N'H); ^13^C-NMR (CDCl_3_) δ: 14.6 (CH_3_), 22.8 (CH_2_), 25.7 (CH_2_), 29.1 (CH_2_), 29.3 (CH_2_), 29.5 (CH_2_ × 2), 31.9 (CH_2_), 37.5 (CH_2_), 120.1 (C-3', C-5'), 122.7 (C-3), 123.8 (C-5), 123.9 (C-1), 125.6 (C-1'), 129.4 (C-6), 130.8 (C-2', C-6'), 132.3 (C-4), 138.3 (C-2), 142.9 (C-4’), 167.7 (CON'H, COOH), 171.8 (NHCO); GC-MS (EI) *m/z*: 273 [M−1−C_7_H_6_NO_2_]^+^.

*2-Decanoylamino-1-(3-carboxyphenyl)benzamide* (**13**). The crude compound was recrystallized with ethyl acetate/*n*-hexane = 1:2 mixture to give the pure compound as a white powder. Yield: 51%; mp: 167–168 °C; ^1^H-NMR (CDCl_3_) δ: 0.78 (3H, t, *J* = 6.4 Hz, CH_3_), 1.15–1.19 (12H, m, CH_2_ × 6), 1.52 (2H, quint, *J* = 13.6, 6.8 Hz, CH_2_), 2.26 (2H, t, *J* = 7.2 Hz, CH_2_), 7.17 (1H, dd, *J* = 7.6, 7.4 Hz, H-5), 7.31 (1H, dd, *J* = 7.8, 7.8 Hz, H-5'), 7.47 (1H, dd, *J* = 7.8, 7.8 Hz, H-4), 7.74 (2H, d, *J* = 8.8 Hz, H-4', H-6'), 7.78 (1H, d, *J* = 7.6 Hz, H-6), 8.17 (1H, d, *J* = 8.4 Hz, H-3), 8.38 (1H, s, H-2'), 10.51 (1H, s, N'H), 10.53 (1H, s, NH); ^13^C-NMR (CDCl_3_) δ: 14.6 (CH_3_), 22.7 (CH_2_), 25.6 (CH_2_), 29.2 (CH_2_), 29.3 (CH_2_), 29.4 (CH_2_), 29.5 (CH_2_), 31.9 (CH_2_), 37.7 (CH_2_), 122.2 (C-2'), 122.7 (C-3), 123.6 (C-5), 123.9 (C-4'), 124.4 (C-6'), 126.0 (C-1, C-1'), 128.5 (C-6), 129.4 (C-5'), 132.3 (C-4), 138.8 (C-2), 139.0 (C-3'), 167.7 (CON'H, COOH), 171.8 (NHCO); GC-MS (EI) *m/z*: 273 [M−1−C_7_H_6_NO_2_]^+^.

*2-Dodecanoylamino-1-(4-carboxyphenyl)benzamide* (**14**). The crude compound was column chromatographed with ethyl acetate/*n*-hexane = 1:1 mixtures and 10% MeOH in dichloromethane to give the pure compound as a white powder. Yield: 58%; mp: 159–160 °C; ^1^H-NMR (CDCl_3_) δ: 0.80 (3H, t, *J* = 6.8 Hz, CH_3_), 1.17–1.20 (16H, m, CH_2_ × 8), 1.51 (2H, quint, *J* = 13.6, 6.8 Hz, CH_2_), 2.26 (2H, t, *J* = 7.2 Hz, CH_2_), 7.18 (1H, dd, *J* = 7.6, 7.6 Hz, H-5), 7.48 (1H, dd, *J* = 8.0, 7.8 Hz, H-4), 7.72 (1H, d, *J* = 8.4 Hz, H-6), 7.74 (2H, d, *J* = 8.8 Hz, H-3', H-5'), 7.92 (2H, d, *J* = 8.4 Hz, H-2', H-6'), 8.06 (1H, d, *J* = 8.0 Hz, H-3), 10.35 (1H, s, NH), 10.60 (1H, s, N'H); ^13^C-NMR (CDCl_3_) δ: 14.6 (CH_3_), 22.8 (CH_2_), 25.7 (CH_2_), 29.2 (CH_2_), 29.4 (CH_2_), 29.5 (CH_2_), 29.6 (CH_2_ × 3), 31.9 (CH_2_), 37.5 (CH_2_), 120.0 (C-3', C-5'), 122.7 (C-3), 123.8 (C-5), 123.9 (C-1), 125.4 (C-1'), 129.5 (C-6), 130.8 (C-2', C-6'), 132.3 (C-4), 138.3 (C-2), 142.3 (C-4'), 167.7 (CON'H, COOH), 171.9 (NHCO); GC-MS (EI) *m/z*: 301 [M−1−C_7_H_6_NO_2_]^+^.

*2-Dodecanoylamino-1-(3-carboxyphenyl)benzamide* (**15**). The crude compound was column chromatographed with ethyl acetate/*n*-hexane = 1:1 mixtures and 10% MeOH in dichloromethane to give the pure compound as a white powder. Yield: 69%; mp: 185–187 °C; ^1^H-NMR (CDCl_3_) δ: 0.80 (3H, t, *J* = 6.8 Hz, CH_3_), 1.16–1.21 (16H, m, CH_2_×8), 1.51 (2H, quint, *J* = 14.4, 7.2 Hz, CH_2_), 2.27 (2H, t, *J* = 7.4 Hz, CH_2_), 7.18 (1H, dd, *J* = 7.6, 7.6 Hz, H-5), 7.35 (1H, dd, *J* = 8.0, 7.8 Hz, H-5'), 7.48 (1H, dd, *J* = 8.4, 1.0 Hz, H-4), 7.69 (1H, s, H-6'), 7.76 (1H, d, *J* = 7.6 Hz, H-6), 7.81 (1H, d, *J* = 6.8 Hz, H-4'), 8.13 (1H, d, *J* = 8.0 Hz, H-3), 8.34 (1H, s, H-2'), 10.47 (1H, s, NH), 10.49 (1H, s, N'H); ^13^C-NMR (CDCl_3_) δ: 14.6 (CH_3_), 22.8 (CH_2_), 25.7 (CH_2_), 29.2 (CH_2_), 29.4 (CH_2_), 29.6 (CH_2_ × 4), 31.9 (CH_2_), 37.6 (CH_2_), 121.1 (C-2'), 122.4 (C-3), 123.7 (C-5), 123.8 (C-4'), 124.0 (C-6'), 124.8 (C-1), 125.7 (C-1'), 129.4 (C-6), 129.5 (C-5'), 132.3 (C-4), 138.6 (C-2), 139.2 (C-3'), 167.7 (CON'H, COOH), 171.8 (NHCO); GC-MS (EI) *m/z*: 301 [M−1−C_7_H_6_NO_2_]^+^.

*2-Tetradecanoylamino-1-(4-carboxyphenyl)benzamide* (**16**). The crude compound was recrystallized with ethyl acetate/*n*-hexane = 1:1 mixture to give the pure compound as a white powder. Yield: 65%; mp: 178–179 °C; ^1^H-NMR (CDCl_3_) δ: 0.81 (3H, t, *J* = 6.8 Hz, CH_3_), 1.16–1.22 (20H, m, CH_2_ × 10), 1.51 (2H, quint, *J* = 14.0, 7.0 Hz, CH_2_), 2.26 (2H, t, *J* = 7.2 Hz, CH_2_), 7.20 (1H, dd, *J* = 7.6, 7.4 Hz, H-5), 7.48 (1H, dd, *J* = 7.8, 7.8 Hz, H-4), 7.71 (1H, d, *J* = 7.6 Hz, H-6), 7.81 (2H, d, *J* = 8.8 Hz, H-3', H-5’), 7.90 (2H, d, *J* = 8.8 Hz, H-2', H-6'), 8.01 (1H, d, *J* = 8.0 Hz, H-3), 10.25 (1H, s, NH), 10.60 (1H, s, N'H); ^13^C-NMR (CDCl_3_) δ: 14.6 (CH_3_), 22.8 (CH_2_), 25.7 (CH_2_), 29.2 (CH_2_), 29.4 (CH_2_), 29.5 (CH_2_), 29.6 (CH_2_), 29.7 (CH_2_ × 4), 32.0 (CH_2_), 37.4 (CH_2_), 120.2 (C-3', C-5'), 122.9 (C-3), 123.9 (C-5), 125.9 (C-1), 126.9 (C-1'), 129.4 (C-6), 130.8 (C-2', C-6'), 132.3 (C-4), 138.2 (C-2), 143.6 (C-4'), 167.8 (CON'H, COOH), 171.9 (NHCO); GC-MS (EI) *m/z*: 329 [M−1−C_7_H_6_NO_2_]^+^.

*2-Tetradecanoylamino-1-(3-carboxyphenyl)benzamide* (**17**). The crude compound was recrystallized with ethyl acetate/*n*-hexane = 1:2 mixture to give the pure compound as a white powder. Yield: 74%; mp: 184–185 °C; ^1^H-NMR (CDCl_3_) δ: 0.81 (3H, t, *J* = 6.6 Hz, CH_3_), 1.15–1.22 (20H, m, CH_2_ × 10), 1.53 (2H, quint, *J* = 14.4, 7.2 Hz, CH_2_), 2.27 (2H, t, *J* = 7.2 Hz, CH_2_), 7.19 (1H, dd, *J* = 7.6, 7.6 Hz, H-5), 7.41 (1H, dd, *J* = 7.8, 7.8 Hz, H-5'), 7.48 (1H, dd, *J* = 8.0, 7.8 Hz, H-4), 7.66 (1H, d, *J* = 7.6 Hz, H-6'), 7.75 (1H, d, *J* = 7.6 Hz, H-6), 7.89 (1H, d, *J* = 8.0 Hz, H-4'), 8.09 (1H, d, *J* = 8.4 Hz, H-3), 8.33 (1H, s, H-2'), 10.38 (1H, s, NH), 10.50 (1H, s, N'H); ^13^C-NMR (CDCl_3_) δ: 14.6 (CH_3_), 22.8 (CH_2_), 25.7 (CH_2_), 29.2 (CH_2_), 29.4 (CH_2_), 29.5 (CH_2_), 29.7 (CH_2_ × 5), 32.0 (CH_2_), 37.6 (CH_2_), 122.1 (C-2'), 122.5 (C-3), 123.8 (C-5), 124.8 (C-4'), 124.9 (C-6'), 125.1 (C-1), 125.5 (C-1'), 129.3 (C-6), 129.5 (C-5'), 132.4 (C-4), 138.4 (C-2), 139.6 (C-3'), 167.7 (CON'H, COOH), 171.8 (NHCO); GC-MS (EI) *m/z*: 329 [M−1−C_7_H_6_NO_2_]^+^.

*2-Hexadecanoylamino-1-(3-carboxyphenyl)benzamide* (**18**). The crude compound was recrystallized with ethyl acetate/*n*-hexane = 1:2 mixture to give the pure compound as a pale yellow powder. Yield: 64%; mp: 192–193 °C; ^1^H-NMR (CDCl_3_) δ: 0.81 (3H, t, *J* = 6.8 Hz, CH_3_), 1.14–1.22 (24H, m, CH_2_ × 12), 1.51 (2H, quint, *J* = 14.4, 7.2 Hz, CH_2_), 2.26 (2H, t, *J* = 7.2 Hz, CH_2_), 7.19 (1H, dd, *J* = 7.6, 0.8 Hz, H-5), 7.48 (1H, dd, *J* = 7.2, 1.4 Hz, H-4), 7.71 (1H, d, *J* = 8.0 Hz, H-6), 7.80 (2H, d, *J* = 8.4 Hz, H-3', H-5'), 7.90 (2H, d, *J* = 8.4 Hz, H-2', H-6'), 8.03 (1H, d, *J* = 8.0 Hz, H-3), 10.28 (1H, s, NH), 10.60 (1H, s, N'H); ^13^C-NMR (CDCl_3_) δ: 14.6 (CH_3_), 22.8 (CH_2_), 25.7 (CH_2_), 29.2 (CH_2_), 29.4 (CH_2_), 29.6 (CH_2_), 29.7 (CH_2_ × 7), 32.0 (CH_2_), 37.5 (CH_2_), 120.2 (C-3', C-5'), 122.9 (C-3), 123.9 (C-5), 125.8 (C-1), 127.4 (C-1'), 129.4 (C-6), 130.7 (C-2', C-6'), 132.3 (C-4), 138.2 (C-2), 143.3 (C-4'), 167.8 (CON'H, COOH), 171.9 (NHCO); GC-MS (EI) *m/z*: 358 [M−1−C_7_H_6_NO_2_]^+^.

*2-Hexadecanoylamino-1-(3-carboxyphenyl)benzamide* (**19**). The crude compound was column chromatographed with ethyl acetate/*n*-hexane = 1:1 mixtures and 10% MeOH in dichloromethane to give the pure compound as a white powder. Yield: 66%; mp: 164–165 °C; ^1^H-NMR (CDCl_3_) δ: 0.79 (3H, t, *J* = 6.6 Hz, CH_3_), 1.15–1.17 (24H, m, CH_2_ × 12), 1.51 (2H, quint, *J* = 12.8, 6.4 Hz, OCH_2_CH_2_), 2.26 (2H, t, *J* = 7.2 Hz, OCH_2_CH_2_), 7.16 (1H, dd, *J* = 7.6, 7.4 Hz, H-5), 7.31 (1H, dd, *J* = 7.6, 7.6 Hz, H-5'), 7.46 (1H, dd, *J* = 7.8, 7.8 Hz, H-4), 7.69 (1H, d, *J* = 7.2 Hz, H-6'), 7.78 (2H, d, *J* = 7.6 Hz, H-6, H-4'), 8.16 (1H, d, *J* = 8.0 Hz, H-3), 8.34 (1H, s, H-2'), 10.50 (1H, s, N'H), 10.55 (1H, s, NH); ^13^C-NMR (CDCl_3_) δ: 14.6 (CH_3_), 22.8 (CH_2_), 25.7 (CH_2_), 29.2 (CH_2_), 29.4 (CH_2_), 29.6 (CH_2_), 29.7 (CH_2_ × 7), 32.0 (CH_2_), 37.5 (CH_2_), 122.3 (C-2'), 122.5 (C-3), 123.6 (C-5), 123.9 (C-4'), 124.5 (C-6'), 125.6 (C-1), 128.6 (C-1'), 129.4 (C-6), 129.5 (C-5'), 132.3 (C-4), 138.7 (C-2), 139.1 (C-3'), 167.7 (CON'H, COOH), 171.8 (NHCO); GC-MS (EI) *m/z*: 358 [M−1−C_7_H_6_NO_2_]^+^.

### 3.3. PCAF HAT Inhibition Assay

The structural analogues were derived from anthranilic acid by introducing the acyl chain at the 2- position, and 3- or 4-carboxyaniline at the 1-position, respectively. The ability of new compounds **8**–**19** to inhibit HAT activity was analyzed *in vitro* using PCAF HAT enzymatic kit (Cayman Co., Ann Arbor, MI, USA). Anacardic acid, a commercially available HAT inhibitor, was used as a positive control. 

The PCAF HAT inhibition studies were performed using a fluorescent assay as described previously [30,31]. The enzyme activity was measured by the detection of CoA-SH by the fluorescent dye 7-(diethylamino)-3-(4'-maleimidylphenyl)-4-methylcoumarin (CPM). In brief, assay buffer (15 μL), acetyl CoA (5 μL) and diluted PCAF (10 μL) were added to all the wells. Dimethylsulfoxide (DMSO, 5 μL) was added to 100% initial activity wells and the background wells, and inhibitor (100 μM, 5 μL) was added to the inhibitor wells. PCAF HAT peptide (20 μL) was added to all the wells except the background wells. The wells were incubated on a shaker for 5 minutes at room temperature and HAT stop reagent (50 μL) was added to all the wells including the background wells. HAT peptide (20 μL) was added to the background wells only. HAT developer (100 μL) was added to all the wells including the background wells and incubated for 20 minutes at room temperature. Then, read the plate using an excitation wavelength of 360–390 nm and an emission wavelength of 450–470 nm. Subtract the fluorescence of the background wells from the fluorescence of the 100% initial activity and the inhibitor wells. The percent inhibition for each sample was determined as follows:

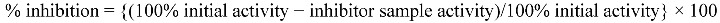


### 3.4. SRB Assay

#### 3.4.1. Cells

The cell lines used were HSF (normal cell), HT-29 (human colon cancer), HCT-116 (human colon cancer), MDA-231 (human breast cancer), A549 (human lung cancer), Hep3B (human hepatoma cancer), HeLa (human cervical cancer) and Caki (human kidney cancer) supplied from the Korean Cell Line Bank (Daejeon, Korea). Cells were grown as monolayer cultures in the T-175 flasks (Costar), were subcultured twice a week at 37 °C in an atmosphere containing 5% CO_2_ in air and 100% relative humidity and maintained at low passage number (5 to 20). RPMI medium 1640, Dulbecco’s modified eagle medium (DMEM, Gibco), fetal bovin serum, (FBS, Gibco), penicillin-streptomycin (P-S, Gibco), phosphate buffered saline pH 7.4 (PBS, Gibco), 0.25% trypsin-EDTA (Gibco), 0.4% trypan blue, dimethyl sulfoxide (DMSO) (Sigma-Aldrich), trichloroacetic acid (Sigma-Aldrich) and sulforhodamine B (SRB) (Sigma-Aldrich) were used.

#### 3.4.2. Cell Inoculation

Adherent cells at the logarithmic growth phase were detached the by an addition of 3 mL of trypsin-EDTA (1:250) mixture, and they were incubated for 5 min at 37 °C. The cells were plated (200 μL per well) in 96 well flat-bottom microplates at densities of 3 × 10^3^ cells per well. The cells were plated in sextuplicate (six replicate wells per cell density) and experiments were performed three times. Microplates were left for 6 h at 37 °C, so that the cells were able to attach to the bottom of the wells before the fixation protocol was carried out. 

#### 3.4.3. Fixation

The culture medium was aspirated prior to fixation. Then, cold (4 °C) 50% TCA (50 μL) was added to the top of 200 μL culture medium in each well to produce a final TCA concentration of 10%. Microplates were left for 30 min at 4 °C, and subsequently, they were washed five times with deionized water. Microplates were then left to dry at room temperature for at least 24 h. 

#### 3.4.4. SRB Assay Protocol

The SRB assay was carried out as previously described [32]. In brief, 0.4% (w/v) sulforhodamine B (70 μL) in acetic acid solution were added to each well and left at room temperature for 20 min. SRB was removed and the plates washed five times with 1% acetic acid before air drying. Bound SRB was solubilized with 10 mM unbuffered Tris-base solution (200 μL) and the plates were left on a plate shaker for at least 10 min. Absorbance was read in a 96-well plate reader at 492 nm subtracting the background measurement at 620 nm. The test optical density (OD) value was defined as the absorbance of each individual well, minus the blank value.

### 3.5. Caco-2 Cell Permeability Test

#### 3.5.1. Caco-2 Cell Culture

The Caco-2 cell line was obtained from the American Type Culture Collection (No. HTB-37, passage #34, Manassas, VA, USA) and Korean Cell Line Bank (No. 30037.1, passage #36). Cells were seeded at 6.8 × 10^4^ cells/cm^2^ on to polycarbonate filter membranes with pore sizes of 0.4 μM and growth areas of 1.1 cm^2^ in clusters of 12 wells (Corning Costar Corp., Cambridge, MA, USA). The cells were grown in a medium consisting of Dulbecco’s modified Eagle’s medium (DMEM) containing 4.5 g/L of fetal bovine serum (FBS), 1% non-essential amino acids (NEAA), 1% L-glutamine, penicillin (100 IU/mL), and streptomycin (100 μg/mL). The clusters were maintained at 37 °C in an incubator in an atmosphere of 5% CO_2_ and 95% air, at 95% relative humidity. The growth medium was changed three times a week until time of use. Cells from passage numbers 31 to 42 were used for the transport experiments. The monolayers were used in experiments at ages ranging from 21 to 28 days. 

#### 3.5.2. Caco-2 Cell Permeability

The transport buffer employed in the transport studies contained 0.01 M of phosphate buffer saline (PBS^+^), which was supplemented with 0.45 M calcium chloride and 0.4 M potassium chloride and adjusted to pH 6.0. Transwell, with Caco-2 cells grown on them for 21 days, were rinsed twice and equilibrated with PBS^+^ transporter buffer at 37 °C for 15 min before the transport experiment. 

Before the permeability experiments, the cell monolayers had been washed twice with HBSS (Hank’s balanced salt solution) containing 10 mM 4-(2-hydroxyethyl)piperazino-1-ethanesulfonic acid (HEPES, pH 7.4). After washing, the cells were allowed to come to equilibrium in the transport buffer for 30 min. Trans-epithelial electrical resistance (TEER) was measured using a EVOM2 equipped with STX2 electrode (World Precision Instruments Inc., Sarasota, FL, USA). Cell monolayers with TEER values below 200 Ω were not used.

The test compounds were first prepared in DMSO solution (10 mM) and then in HBSS buffer to give a final drug concentration of 50 μM when added to the cell monolayers. Samples were obtained after 15, 30, 45, 60, and 90 min by moving the cell monolayers to a new receiver well containing fresh HBSS.

Drug concentrations in the receiver compartments were determined using HPLC (Waters 2690, Milford, CT, USA) and a Waters 2487dual λ absorbance detector. Agilent ZORBAX 300SB-C18 (5 μm, 4.6 × 250 mm) and Agilent ZORBAX SB-C8 (5 μm, 4.6 × 250 mm) were used as HPLC columns with a flow rate of 1.0 mL/min. The mobile phase consisted of 1.1% triethylamine (pH 3.0) and acetonitrile (95:5~40:60). 

## 4. Conclusions

Work is underway in order to clarify the role of the different modifications (3- or 4-carboxybenzamide, chain length, anthranilic acid skeleton) on the PCAF HAT inhibiting activities, cytotoxicity against seven cancer cell lines of HT-29 (colon), HCT-116 (colon), MDA-231 (breast), A549 (lung), Hep3B (hepatoma), HeLa (cervical) and Caki (kidney) and one normal cell line (HSF). 3- or 4-Carboxybenzamide group and acyl chain length were not necessarily proportional to the PCAF HAT inhibitory activities, but a long acyl chain is important to inhibitory activities. The cytotoxicity of **17** (C14) and **18** (C16) is higher than that of anacardic acid against seven cancer cell lines by SRB assay. The two selected compounds **16** and **17** were predicted to have high drug absoption in humans by a Caco-2 cell permeability assay. 

## References

[1] Biel M., Wascholowski V., Giannis A. (2005). Epigenetics-an epicenter of gene regulation: Histones and histone-modifying enzymes. Angew. Chem. Int. Ed. Engl..

[2] Feinberg A.P., Vogelstein B. (1983). Hypomethylation distinguishes genes of some human cancers from their normal counterparts. Nature.

[3] Nakao M. (2001). Epigenetics: Interaction of DNA methylation and chromatin. Gene.

[4] Hake S.B., Xiao A., Allis C.D. (2004). Linking the epigenetic “language” of covalent histone modification to cancer. Br. J. Cancer.

[5] Inche A.G., La thangue N.B. (2006). Chromatin control and cancer-drug discovery: Realizing the promise. Drug Discov. Today.

[6] Davis C.D., Ross S.A. (2007). Dietary components impact histone modifications and cancer risk. Nutr. Rev..

[7] Ghizzoni M., Boltjes A., de Graaf C., Haisma H.J., Dekker F.J. (2010). Improved inhibition of the histone acetyl transferase PCAF by an anacardic acid derivative. Bioorg. Med. Chem..

[8] Carrozza M.J., Utley R.T., Workman J.L., Cote J. (2003). The diverse functions of histone acetyl transferase complexes. Trends Genet..

[9] Sterner D.V., Berger S.L. (2000). Acetylation of histones and transcription-related factors. Microbiol. Mol. Biol. Rev..

[10] Dekker F.J., Haisma H.J. (2009). Histone acetyl transferases as emerging drug targets. Drug Discov. Today.

[11] Lau O.D., Kundu T.K., Soccio R.E. (2000). HATs off: Selective synthetic inhibitors of the histone acetyltransferase p300 and PCAF. Mol. Cell.

[12] Balasubramanyam K., Altaf M., Varier R.A.V., Swaminathan V., Ravindran A., Sadhale P.P., Kundu T.K. (2004). Polyisoprenylated benzophenone, Garcinol, A natural histone acetyltransferase inhibitor, Represses chromatin transcription and alters global gene expression. J. Biol. Chem..

[13] Balasubramanyam K., Varier R.A., Altaf M.V., Swaminathan V., Siddappa N.B., Ranga U., Kundu T.K. (2004). Curcumin, A novel p300/CREB-binding protein-specific inhibitor of acetyltransferase, Represses the acetylation of histone/nonhistone proteins and histone acetyltransferase-dependent chromatin transcription. J. Biol. Chem..

[14] Stimson L., Rowlands M.G., Newbatt Y.M., Smith N.F., Raynaud F.I., Rogers P., Bavetsias V., Gorsuch S., Jarman M., Bannister A. (2005). Isothiazolones as inhibitors of PCAF and p300 histone acetyltransferase activity. Mol. Cancer Ther..

[15] Hemshekhar M., Santhosh M.S., Kemparaju K., Girish K.S. (2012). Emerging roles of anacardic acid and its derivatives: A pharmacological overview. Basic Clin. Pharmacol. Toxcol..

[16] Eliseeva E., Valkov V., Jung M., Jung M.O. (2007). Characterization of novel inhibitors of histone acetyltransferases. Mol. Cancer Ther..

[17] Souto J.A., Conte M., Alvarez R., Nebbioso A., Carafa V., Altucci L., Lera A.R. (2008). Synthesis of benzamides related to anacardic acid and their histone acetyltransferase (HAT) inhibitory activities. ChemMedChem.

[18] Chandregowda V., Kush A., Reddy G.C. (2009). Synthesis of benzamide derivatives of anacardic acid and their cytotoxic activity. Eur. J. Med. Chem..

[19] Mantelingu K., Kishore A.H., Balasubramanyam K., Kumar G.V., Altaf M., Swamy S.N., Selvi R., Das C., Narayana C., Rangappa K.S. (2007). Activation of p300 histone acetyltransferase by small molecules altering enzyme structure: Probed by surface-enhanced Raman spectroscopy. J. Phys. Chem. B.

[20] Sbardella G., Castellano S., Vicidomini C., Rotili D., Nebbioso A., Miceli M., Aitucci L., Mai A. (2008). Identification of long chain alkylidenemalonates as novel small molecule modulators of histone acetyl transferases. Bioorg. Med. Chem. Lett..

[21] Green I.R., Tocoli F.E., Lee S.H., Nihei K., Kubo L. (2007). Molecular design of anti-MRSA agents based on the anacardic acid scaffold. Bioorg. Med. Chem..

[22] Hird N.W., Milner P.H. (1994). Synthetic and β-lactamase inhibition of anacardic acids and their analogues. Bioorg. Med. Chem. Lett..

[23] Yamagiwa Y., Ohashi K., Sakamota Y., Hirakawa S., Kamikawa T., Kubo I. (1987). Syntheses of anacardic acids and ginkgoic acid. Tetrahedron.

[24] Levin J.I., Turos E., Weinreb S.M. (1982). An alternative procedure for the aluminium-medated conversion of esters to amides. Syn. Commun..

[25] Gustafsson T., Ponten F., Seeberger P.H. (2008). Trimethylaluminum mediated amide bond formationin a continuous flow microreactor as key to the synthesis of rimonabant and efaproxiral. Chem. Commun..

[26] Sambuy Y., De Angelis I., Ranaldi G., Scarino M.L., Stammati A., Zucco F. (2005). The Caco-2 cell line as a model of the intestinalbarrier: Influence of cell and culture-related factors on Caco-2 cellfunctional characteristics. Cell Biol. Toxicol..

[27] Azenha M.A., Evangelista R., Martel F., Vasconcelos M.T. (2004). Estimation of the human intestinal permeability of butyltin speciesusing the Caco-2 cell line model. Food Chem. Toxicol..

[28] Artursson P., Palm K., Luthman K. (1996). Caco-2 monolayers in experimental and theoretical predictions of drug transport. Adv. Drug Deliv. Rev..

[29] Artursson P., Karlsson J. (1991). Correlation between oral drug absorption in humans and apparent drug permeability coefficients in human intestinal epithelial (Caco-2) cells. Biochem. Biophys. Res. Commun..

[30] Dekker F.J., Ghizzoni M., van der Meer N., Wisastra R., Haisma H.J. (2009). Inhibition of the PCAF histone acetyltransferase and cell proliferation by isothiazolones. Bioorg. Med. Chem..

[31] Trievel R.C., Li F.Y., Marmorstein R. (2000). Application of a fluorescent histone acetyltransferase assay to probe the substrate specificity of human p300/CBP-associated factor. Anal. Biochem..

[32] Skehan P., Storeng R., Scudiero D., Monks A., McMahon J., Vistica D., Warren J.T., Bodesh H., Kenny S., Boyd M.R. (1990). New colorimetric cytotoxicity assay for anticancer-drug screening. J. Natl. Cancer Inst..

